# Strategies for programmable manipulation of alternative splicing

**DOI:** 10.1016/j.gde.2024.102272

**Published:** 2024-10-29

**Authors:** Jonathan C Schmok, Gene W Yeo

**Affiliations:** 1 Department of Cellular and Molecular Medicine, University of California San Diego, La Jolla, CA, USA; 2 Sanford Stem Cell Institute Innovation Center and Stem Cell Program, University of California San Diego, La Jolla, CA, USA; 3 Institute for Genomic Medicine, University of California San Diego, La Jolla, CA, USA; 4 UCSD Center for RNA Technologies and Therapeutics, University of California San Diego, La Jolla, CA, USA; 5 Department of Bioengineering, University of California, San Diego, La Jolla, CA, USA

## Abstract

Alternative splicing (AS) plays a pivotal role in protein diversity and mRNA maturation. Programmable control of targeted AS events is of longstanding interest in RNA biology, promising correction of dysregulated splicing in disease and discovery of AS events. This review explores four main strategies for programmable splicing manipulation: (1) inhibiting splicing signals with antisense oligonucleotides (ASOs), exemplified by therapies approved by the U.S. Food and Drug Administration, (2) applying DNA-targeting clustered regularly interspaced short palindromic repeats systems to edit splicing signals, (3) using synthetic splicing factors, including synthetic proteins and ribonucleoproteins, inspired by natural RNA-binding proteins, and (4) guiding endogenous splicing machinery with bifunctional ASOs and engineered small nuclear RNAs. While ASOs remain clinically prominent, emerging technologies aim for broad, scalable, durable, and precise splicing modulation, holding promise for transformative advancements in RNA biology and therapeutic interventions.

## Introduction

Splicing is a key regulatory step in the maturation of mRNA, orchestrated by a complex process led by the spliceosome, a macromolecular machine consisting of multiple ribonucleoprotein (RNP) particles. When RNA signals at and near exon–intron boundaries are recognized, spliceosomal RNPs bind to these signals and initiate an enzymatic cascade that removes introns and joins exons. In alternative splicing (AS), certain exons are included at characteristic frequencies dictated by multiple factors, such as cell type, environmental cues, disease states, and genetic variation. Recent estimates propose that 92–94% of protein-coding genes produce multiple splicing isoforms [60]. Aberrant splicing outcomes are widespread in disease, presenting potential therapeutic targets [24]. At alternatively spliced exons, the RNA signals defining the exon–intron boundaries exhibit degeneracy, reducing the binding affinity of spliceosomal RNPs. The detection and inclusion of alternatively spliced exons are primarily regulated by splicing factors (SFs), a class of RNA-binding proteins (RBPs) that bind to signals on or nearby alternatively spliced exons and can enhance or inhibit spliceosomal recognition of the exon, resulting in increased or reduced exon inclusion, respectively.

**Figure 1:**
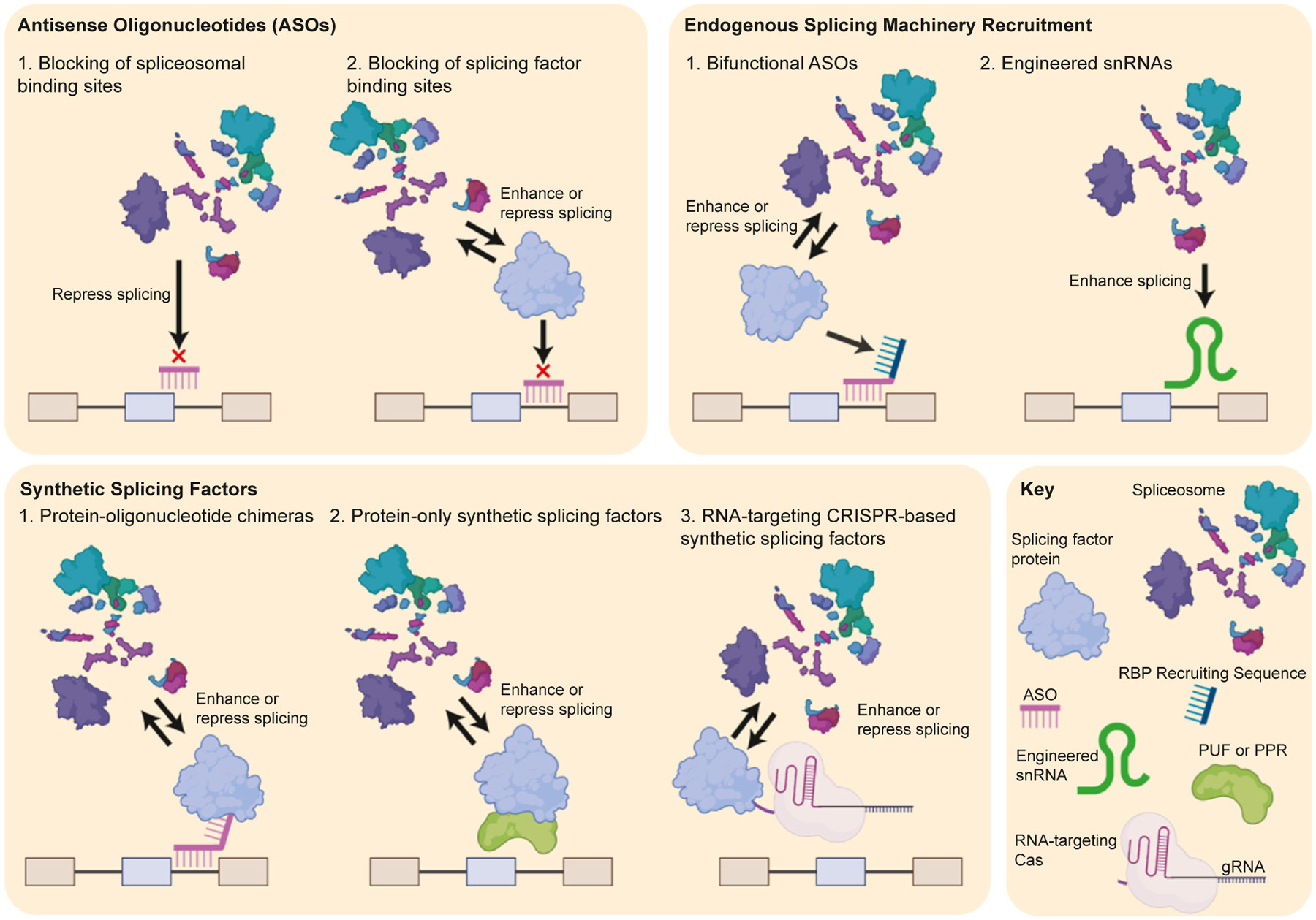
Overview of RNA-targeting technologies used for programmable manipulation of AS. Current RNA-targeting technologies used to programmably manipulate AS can be summarized into three main categories: ASOs, endogenous splicing machinery recruitment, and synthetic SFs. ASOs act through two primary mechanisms of action: (1) the blocking of spliceosomal binding sites, repressing splicing of an exon and (2) the blocking of SF sites, inhibiting the action of said SF, which can enhance or repress exon inclusion. Two techniques used in endogenous splicing machinery recruitment are bifunctional ASOs and engineered snRNAs. Bifunctional ASOs consist of an antisense region, which binds to the target of interest and a splicing machinery recruitment or repression region. Engineered snRNAs appear to the spliceosome as a normal component of the spliceosomal machinery but are tuned to recognize their target at higher affinity, inducing exon inclusion. Three types of artificial SFs are protein-oligonucleotide chimeras, protein-only synthetic SFs, and RNA-targeting CRISPR-based synthetic SFs. Protein-oligonucleotide chimeras consist of an oligonucleotide region that binds antisense to the target, fused to a protein that can enhance or repress exon inclusion. Protein-only synthetic SFs perform both the binding and the effector function with protein domains. RNA-targeting CRISPR-based synthetic SFs consist of an effector domain fused to an RNA-targeting Cas protein, that associates with a co-expressed guide RNA to bind the target of interest.

The foundation of synthetic biology lies in harnessing natural biological processes for useful purposes. Since the initial discovery and characterization of AS, researchers have sought to develop tools and methods for controlling splicing outcomes in scientific and therapeutic contexts. Gaining direct control over the inclusion level of any targeted exon would enable precise probing of its function in a capacity not provided by traditional observational methods. To this point, no unified strategy allows for the arbitrary adjustment of any exon’s inclusion level in either direction. Nonetheless, researchers have applied a diverse range of approaches in various applications, and initial splicing modulation therapies are now available clinically. These strategies can be categorized into four main groups: (1) inhibiting splicing signals with antisense oligonucleotides (ASOs), (2) modulating AS with clustered regularly interspaced short palindromic repeats (CRISPR)-based DNA editing, (3) using synthetic SFs to regulate splicing outcomes, and (4) guiding the endogenous splicing machinery to modify splicing outcomes (RNA-targeting approaches illustrated in Figure 1).

## Inhibiting splicing signals with antisense oligonucleotides

The breadth of applications in which ASOs have been used for splicing modulation is reviewed elsewhere in greater detail [29]. ASOs are 15–30 base-pair DNAs or RNAs, chemically modified for increased binding affinity and stability, that bind to target sequences via Watson-Crick base pairing [44]. ASOs that modulate RNA splicing through steric inhibition of splicing signals are known as splice-switching oligonucleotides (SSOs). The FDA has approved three SSOs: eteplirsen, golodirsen, and nusinersen. Eteplirsen and golodirsen are used in the treatment of Duchenne muscular dystrophy. Both act by inducing skipping of specific exons, restoring the reading frame of *DMD* in patients with frameshift mutations [4,30,35]. Nusinersen is used in the treatment of spinal muscular atrophy and induces inclusion of *SMN2* Exon 7 through steric blocking of an intronic splicing silencer downstream of the exon, leading to increased production of full-length and functional SMN protein from *SMN2* [14]. These three therapies are, to this point, the only clinically approved treatments that act through the modulation of a specific exon using a programmable strategy.

SSOs are effective, relatively safe, and well-validated tools for modulating exon inclusion levels but have some limitations both scientifically and therapeutically. Therapeutically, these SSOs, with extensive modifications, must be delivered directly and cannot be genetically encoded. As the SSOs are eventually cleared, repeated delivery is necessary for the entire clinical duration and involves invasive injections to the affected tissue [39]. Furthermore, many exon targets may not have splicing signals amenable to steric inhibition by SSOs. Though exon skipping can usually be achieved through steric blocking of splice sites, exon inclusion by steric inhibition requires well-defined and strong silencing *cis-*regulatory sequences. Even for exons that have been successfully modulated, extensive searches have been required to find effective SSOs, rendering the approach poorly scalable for screening large panels of exons. The competing technologies discussed below aim to address these limitations.

## Modulating alternative splicing with CRISPR-based DNA editing

While this review primarily focuses on RNA-targeting approaches for splicing modulation, it is also important to acknowledge significant advances in using CRISPR-Cas9 to manipulate splicing signals at the DNA level. An early demonstration of Cas9 gene editing’s impact on AS was an unexpected outcome of β-catenin editing, where unintentionally induced Exon 3 skipping led to nuclear accumulation [38]. Since this discovery, specific genome editing strategies have evolved, such as using base editors to disrupt exon–intron junctions [23,31] and signals bound by trans-acting SFs [2,3,43]. Additionally, dual-gRNA strategies have enabled the excision of alternative exon sequences, demonstrated by both hybrid Cas9-Cas12a platforms [25], and a system expressing two Cas9 gRNAs under separate promoters [57]. These were successfully applied in high-throughput functional exon screening. These genome editing methods offer the advantage of relative permanence from a single treatment when compared with transient RNA-targeting methods, which may be beneficial in therapeutic contexts. However, this permanence also raises concerns about potential genotoxicity of permanent off-target effects and the inability to reverse or adjust the edits based on response.

## Regulating splicing outcomes with synthetic splicing factors

Engineering synthetic RBPs that selectively bind to specific sequences to influence splicing outcomes is an intuitive strategy, considering that RBPs heavily influence endogenous splicing events. Current approaches for engineering RBPs have been largely inspired by the development of engineered DNA-binding proteins, notably synthetic transcriptional activators that can be designed for any DNA target with software [36]. Engineered RBPs are built upon the concept of RBP modularity, which describes RBPs as fusions of targeting domains and effector domains responsible for enzymatic processing of targets [53]. The task of designing an SF can be reduced to selecting these two components: (1) the targeting domain that will specifically recognize the target RNA sequence and (2) the effector domain that will induce or silence exon inclusion as desired. Researchers have designed and tested protein-only synthetic SFs and synthetic RNPs, both of which are covered here. This section is separated by the different approaches used for transcript targeting that have been used in the design of engineered SFs.

### Protein-oligonucleotide chimeras as synthetic splicing factors

The first attempts to engineer synthetic SFs with designed endogenous target specificity and activity were performed using chimeric systems utilizing an antisense-targeting oligonucleotide as the targeting domain, covalently linked to a minimal synthetic exon activating protein domain extracted from the SR protein family of splicing activators [10]. The authors titled their approach ESSENCE (for exon-specific splicing enhancement by small chimeric effectors). This approach was initially demonstrated for correcting aberrant exon skipping in the SMN2 and BRCA1 genes *in vitro*. ESSENCE has also been used to shift the ratio between the bcI-xS and bcI-xL isoforms of the bcl-x gene [66]. Although this approach has been largely supplanted by the other ASO-based strategies discussed in this review that are generally simpler to synthesize and prototype, it remains an important tool to consider in head-to-head comparisons when optimizing targeted exon modulation.

### Protein-only synthetic splicing factors

Researchers have designed protein-only units that perform both targeting and splicing modulatory effector functions, mimicking endogenous human SFs. Many efforts have been inspired by the Pumilio family (PUF) of RBPs. PUFs contain a targeting domain of eight 36-amino acid protein modules that each bind to a single nucleotide [61,65]. Module specificity is driven by three amino acids at characteristic positions that can be swapped to alter the sequence targeted by engineered PUFs [12]. Efforts to build PUF-based artificial SFs have utilized fusions to both exon activation and silencing domains [62]. The authors successfully induced exon inclusion and skipping in splicing reporters and demonstrated exon skipping of endogenous bcl-x. However, they did not demonstrate inclusion of an endogenous exon. Modular design principles of engineered PUF proteins have since been further established, allowing the targeting of sequences longer than the 8 RNA bases of the natural human PUF proteins and the targeting of cytosine, which was unavailable at the time, expanding the potential applicability of this approach [1,19].

Designer proteins based on other modular domains, such as those from pentatricopeptide (PPR) containing proteins and zinc finger (ZF) proteins, are other possibilities for synthetic SFs. PPRs bind RNA with a similar scaffold to PUF proteins, with between 2 and 30 modules of 35 amino acid–binding individual nucleotides specifically [5]. Engineered PPRs have been used to inhibit reporter and endogenous exon inclusion through steric hindrance [67], but PPR and splicing-effector fusion proteins have yet to be evaluated. Preliminary efforts to engineer ZF-based SFs have been inspired by the muscleblind-like (MBNL) family of natural ZF SFs [27]. The authors replaced the two distinct ZF targeting domains in MBNL proteins with heterogeneous duplicates, leading to changes in MBNL binding and activity. Although the rules of RNA recognition by ZFs remain to be established, this work is a promising first step toward synthetic ZF SFs. Altogether, these protein-only approaches offer simplicity and close resemblance to endogenous splicing regulation; however, they struggle with rapid, large-scale redesign and testing compared to methods using antisense base complementarity.

### RNA-targeting CRISPR-based artificial splicing factors

CRISPR-Cas technologies present an adaptable and programmable strategy for targeting nucleic acids, allowing modulation of various cellular processes, including AS of RNA. CRISPR-Cas originates in prokaryotic immune systems as an adaptive response to phage invaders [6]. Initially, the commonly applied DNA-targeting Cas9 system was adapted to target RNA [41]. Concurrently, CRISPR systems that naturally target RNA, such as the Cas13 family of systems, were identified bioinformatically and engineered [52,55,68]. Applications of these systems for modulating AS were hypothesized soon after their emergence [56]. Cas13d was first used for transcriptome engineering by Konermann et al., where it was rendered catalytically dead and used to induce exon skipping through steric hindrance alone, and enhanced through fusion to the glycine-rich domain of hnRNPA1 [32]. Efforts are underway to refine gRNA design guidelines for effective exon skipping using dCas13 steric hindrance, with a recent study successfully inducing exon skipping in 10/13 targets through a systematic approach to gRNA selection utilizing the web-based software Cas13design [26,40,64]. dCas13 steric hindrance has also recently been used to discover new splicing regulatory elements by gRNA tiling [47]. The fusion of exon inclusion activating domains to dCas13d, referred to as CasFx (CRISPR Artificial Splicing Factors), has also been implemented [20]. These have been used as a scientific tool in examining the cross-regulation of SFs in the SRSF family [33]. Initial CasFx systems used established SFs as activation domains, with gRNA position informed by the known rules of SF activity. For instance, RBFOX1-based CasFx systems were used to induce exon inclusion when targeted to the downstream intron [20]. Researchers have iterated upon this strategy by testing various candidates and selecting activation domains for improved potency, reduced size, and reduced binding position dependence.

Approaches have used both MS2-MCP-based tethering screens [51] and screens of dCas13-SF fusions [34], the latter of which led to the most successful CasFx system to date: dCasRx-RBM25, identified as an efficient activator of > 90% of targeted exons. An additional advantage to dCas13-based splicing modulation is the ability of dCas13 enzymes to process arrays of gRNAs, allowing for multiplexed targeting [20]. Analogous systems to RNA-targeting CRISPR derived from human parts have also been developed and could be used similarly [46]. CRISPR-Cas-based SF tethering may hold the greatest promise as a technology for generalized, reprogrammable splicing modulation. Overcoming challenges in significantly increasing exon inclusion and demonstrating broad applicability across various exons have been a significant hurdle; however, the latest iterations of CasFx systems have demonstrated impressive performance.

## Endogenous splicing machinery recruitment

For therapeutic and *in vivo* applications, minimally perturbing cells to achieve the desired outcome is generally favorable. Exogenously expressed proteins, especially those originating in other organisms, raise immunogenicity concerns [13]. Furthermore, introducing new factors into the cellular environment raises the possibility of widespread off-target effects [15,70]. In response to these concerns, methods have emerged to recruit the endogenous splicing machinery expressed in cells to targeted transcripts.

### Recruitment of endogenous splicing machinery with bifunctional ASOs

ASO-based approaches have been used to recruit endogenous SFs to endogenous targets for both activation and repression of exon inclusion. These systems are fully constructed from nucleic acids in contrast to the nucleic acid–protein chimera described earlier [9,54]. These bifunctional systems consist of an RNA-targeting part, complementary to the targeted sequence, and an SF recruiting part. Bifunctional ASO-based exon skipping was first demonstrated for diminishing the bcl-xL/bcl-xS ratio in cultured cancer cells by carrying the binding sites for hnRNP A1/A2 in the SF recruiting part [59]. Bifunctional ASOs have been demonstrated to induce SMN2 Exon 7 directly through enhancement of SMN2 Exon 7 recognition and indirectly through repression of Exon 8 [7,18]. Researchers have further advanced design principles of bifunctional ASOs by demonstrating that strength depends on the number, sequence, and chemistry of SF recruiting motifs [42]. The Chabot lab has developed an algorithmic approach for the design of exon-silencing bifunctional ASOs with an 80% success rate [8]. A similar approach has also been applied using an engineered U7 small nuclear RNA (snRNA) as the bifunctional oligo, which has the advantage of compatibility with transgenic expression [37]. Another potential approach for driving exon skipping was unveiled following the discovery that ASOs with 2′-deoxy-2′-fluoro (2′-F) nucleotides form duplexes with RNAs that specifically recruit the exon skipping proteins ILF2 and ILF3, allowing the development of bifunctional ASOs through chemical modification [48].

### Recruitment of endogenous splicing machinery with engineered small nuclear RNAs

As spliceosomal initiation is driven by snRNAs that recognize the boundaries between exons and introns, enhancing the precision of this process through the development of engineered snRNAs represents a promising strategy for modulating AS. Engineered snRNAs can be developed with increased specificity for a specific target to enhance the natural recognition of that exon and recruit the spliceosome at a higher rate than would naturally occur. The U1 snRNA of the spliceosome has been engineered to increase AS of specified targets [22,28,49]. One caveat in modulating spliceosome association to a transcript via engineered snRNAs is their potential impact on transcript stability. In a study comparing U1 and ASO modulation of SMN2 Exon 7 splicing, U1 increased SMN2 mRNA levels (threefold) and Exon 7-containing pre-mRNA (1.5-fold), effects not observed with ASO [17]. This effect may be beneficial or detrimental, depending on the context, and should be considered when employing engineered snRNAs in both clinical and scientific applications. Additionally, U7 snRNAs, associated with histone pre-mRNA processing, can be used for steric inhibition of splicing signals, similarly to ASOs [45]. Unlike ASOs, which require repeated delivery, custom U7 snRNAs can be delivered via viral vectors for continuous expression. These engineered snRNAs have the advantages of human origin to minimize immune response concerns and to allow compatibility with gene therapy for delivery and, further, are expressed in a high-abundance environment of endogenous snRNAs, reducing concerns of off-target or indirect effects.

## Conclusion

**Figure 2:**
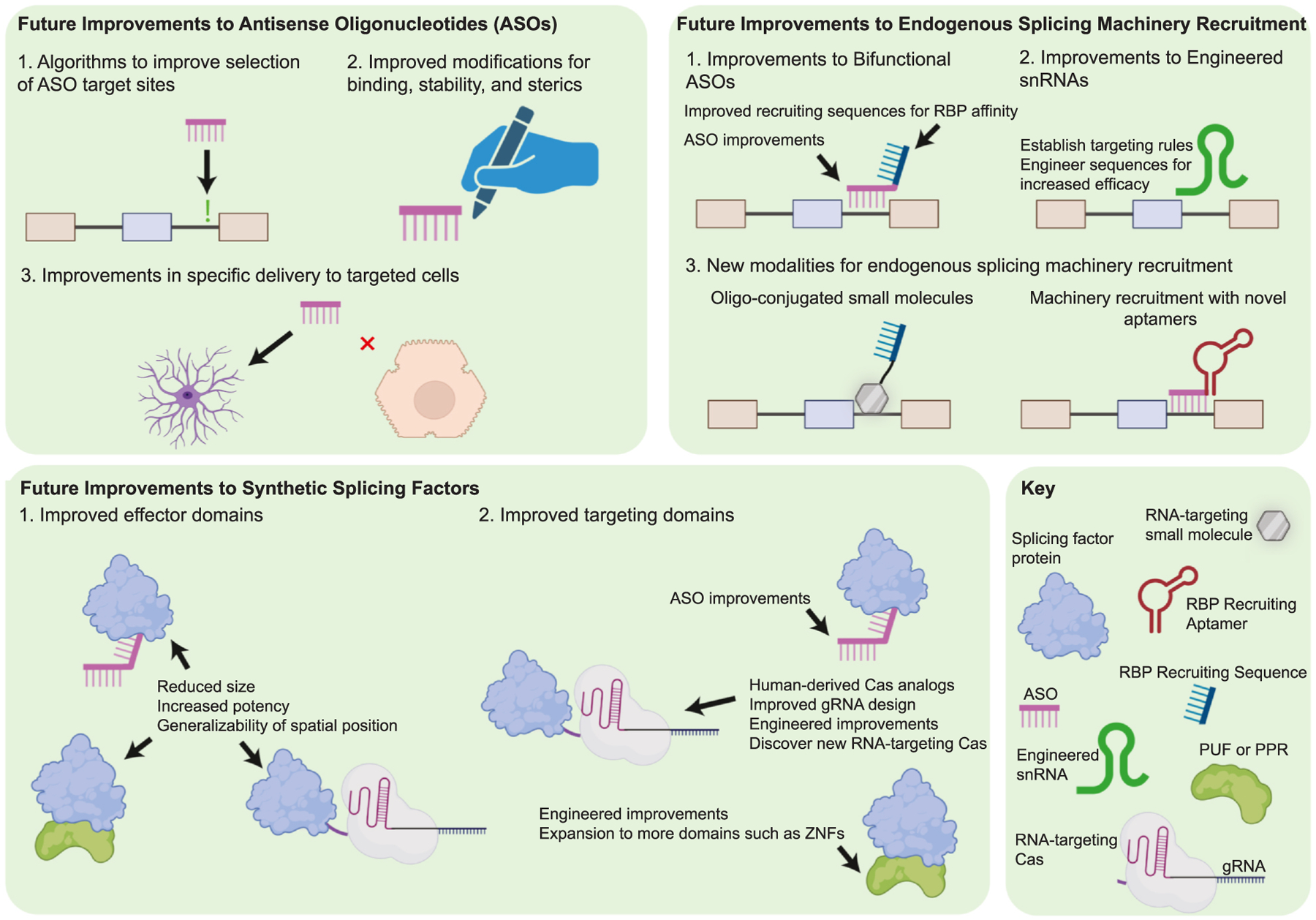
Potential improvements to RNA-targeting technologies used for programmable manipulation of AS. Each category of current technology for programmable manipulation of AS presents opportunities for advancement. Tools and algorithms that generate ASO sequences to manipulate targeted exons have allowed for the rapid development and deployment of ASO therapies to rare diseases. The composition of ASOs can also be improved, with base and backbone modifications to improve binding, stability, and sterics, alongside improvements in delivery techniques for tissue-specific delivery. For endogenous splicing machinery recruitment, both bifunctional ASOs and engineered snRNAs present opportunities for improvement in design, binding efficiency, and effector strength. Additionally, new modalities are in development to recruit endogenous splicing machinery, including oligo-conjugated small molecules and the development of novel RNA aptamers. Synthetic SFs can be improved through improvements of effector domains: specifically size reduction, potency improvement, and generalizability of spatial position relative to targeted exons. They can also be improved through the enhancement of targeting domains for improved binding efficiency, reduced size, and reduced immunogenicity.

The ability to arbitrarily modulate the outcome of splicing events has been an ambitious goal in RNA biology for decades, and several modalities approaching this objective have been introduced. ASOs designed to sterically hinder splicing regulatory regions have long been the standard and, in a few cases, have been clinically approved. Competing approaches to modulate endogenous splicing events through synthetic SFs or recruitment of endogenous SFs have also been introduced and show tremendous promise. However, technical limitations have prevented any of these from being applied in a widespread, generalized way. ASOs appear well-posed to continue as the standard approach for splicing modulation in the clinic, and progress has been made regarding their key limitations, notably in the lead time required to find and test relevant regulatory signals for every targeted splicing event. Tools that algorithmically design ASOs for a targeted splicing event are commercially available, and due to these advances, the nonprofit *n-*Lorem aims to rapidly develop individualized ASOs for patients with rare genetic diseases amenable to splicing modulation therapy [16]. Improvements continue to be made for the competing approaches (Figure 2). CRISPR-Cas9 DNA editing has been used for large-scale functional exon screening and offers potential therapeutic applications as a single-dose effector. The development of RNA-targeting CRISPR systems continues to evolve dynamically, with new variants offering benefits such as reduced [58,63] and enhanced [69] bystander cleavage, as well as miniaturization [71]. Although CasFx systems use catalytically dead Cas13 mutants, making bystander cleavage impossible, advances in miniaturization and potential new variants of Cas13 that improve binding affinity could enhance CasFx technologies. New modalities are also in development for recruiting endogenous splicing machinery, including the development of RNA-targeting small molecules for target selection, and the engineering of novel aptamers to recruit splicing machinery that bind to spliceosomal components due to their higher-order structure [11,21]. With all RNA-targeting methodologies, it is important to understand how RNA secondary structure affects the success of each approach at specific positions. The reliability of ASOs targeting splice junctions to induce exon skipping may alleviate concerns with this method, but a deeper understanding of secondary structures is necessary when targeting splicing silencers and enhancers or using other modulators. Furthermore, understanding the secondary structure of modulator-target interactions is also essential to assess potential off-target effects, as certain structures may permit imperfect matches [50]. Unlocking the ability to tweak any chosen splicing event precisely and dependably for exon function screening is a tantalizing pursuit, promising to reshape the landscape of RNA biology and deepen our grasp of splicing’s intricacies.

## Data Availability

No data were used for the research described in the article.
